# Digital Brain Maps and Virtual Neuroscience: An Emerging Role for Light-Sheet Fluorescence Microscopy in Drug Development

**DOI:** 10.3389/fnins.2022.866884

**Published:** 2022-04-20

**Authors:** Johanna Perens, Jacob Hecksher-Sørensen

**Affiliations:** Gubra ApS, Hørsholm, Denmark

**Keywords:** light-sheet, iDISCO, tissue clearing, obesity, Alzheimer’s disease, Parkinsion’s disease, neurodegeneration, LSFM

## Abstract

The mammalian brain is by far the most advanced organ to have evolved and the underlying biology is extremely complex. However, with aging populations and sedentary lifestyles, the prevalence of neurological disorders is increasing around the world. Consequently, there is a dire need for technologies that can help researchers to better understand the complexity of the brain and thereby accelerate therapies for diseases with origin in the central nervous system. One such technology is light-sheet fluorescence microscopy (LSFM) which in combination with whole organ immunolabelling has made it possible to visualize an intact mouse brain with single cell resolution. However, the price for this level of detail comes in form of enormous datasets that often challenges extraction of quantitative information. One approach for analyzing whole brain data is to align the scanned brains to a reference brain atlas. Having a fixed spatial reference provides each voxel of the sample brains with x-, y-, z-coordinates from which it is possible to obtain anatomical information on the observed fluorescence signal. An additional and important benefit of aligning light sheet data to a reference brain is that the aligned data provides a digital map of gene expression or cell counts which can be deposited in databases or shared with other scientists. This review focuses on the emerging field of virtual neuroscience using digital brain maps and discusses some of challenges incurred when registering LSFM recorded data to a standardized brain template.

## Historical Perspective

Optical imaging of immunolabelled and cleared samples began nearly two decades ago and was initially focused on mouse embryos and smaller organisms (Sharpe et al., 2002; Huisken et al., 2004). However, methods were soon developed to allow imaging of whole organs from adult mice (Alanentalo et al., 2007; Dodt et al., 2007). Since then, a steadily growing number of methods and clearing protocols have been developed for improving biocompatibility, antibody penetration, tissue transparency, and preservation of endogenous fluorescence from proteins like green fluorescent protein (GFP) (Hama et al., 2011; Ertürk et al., 2012; Chung et al., 2013; Renier et al., 2014; Susaki et al., 2014; Klingberg et al., 2017; Ueda et al., 2020). By now the ability to study biology at the whole organ level with single cell resolution has been applied to all mouse organs (Yang et al., 2014; Pan et al., 2016). However, it was immediately recognized that the brain is particularly well-suited for whole organ imaging and consequently, there is a great hope that the light-sheet fluorescence microscopy (LSFM) can help advance our understanding of neuroscience and to aid the development of new drugs and therapies against diseases originating in the central nervous system (CNS). Many disorders that are burdening health care systems around the world have a neuronal origin and therapies focused on treatment of neurodegenerative diseases such as Alzheimer’s disease (AD), Parkinson’s disease (PD), multiple sclerosis (MS), amyotrophic lateral sclerosis (ALS), and Huntington’s disease (HD) are directed towards the brain (Chou et al., 2021). In addition, there is growing evidence that treatment of metabolic diseases such as obesity and diabetes would benefit from a better understanding of the brain (Gasser et al., 2017; Müller et al., 2021). While whole brain imaging of cleared brains has been used to study both the pathology and drug effects in some of the above-mentioned diseases (Secher et al., 2014; Liebmann et al., 2016; Salinas et al., 2018; Detrez et al., 2019; Gabery et al., 2020; Boland et al., 2021; Hansen et al., 2021), LSFM has yet to become an established method in drug development. A possible explanation for this is the relatively low throughput imposed by the enormous datasets generated. The giga- or even terabyte amounts of data generated for each brain that is scanned challenges quantification of desired endpoints. The pharma industry selects and qualifies drugs based on statistical validation of their target, and consequently, high throughput and large study groups are needed. To accommodate this, several parameters need to be optimized: (1) high volume processing of the samples, (2) microscopes and IT infrastructure to enable fast scanning and handling of data, and (3) cloud-based platforms to convey the data generated. In this context, the brain has several advantages over other organs. Due to its immense complexity, the morphology of the mouse brain is very conserved and the brains from two individual mice are almost identical. For this reason, it is possible to align individual mouse brains to a brain template of a reference atlas (Renier et al., 2016; Murakami et al., 2018; Salinas et al., 2018; Wang et al., 2020; Perens et al., 2021a; Newmaster et al., 2022), and at the same time extract spatial information, volumetric information, number of detected cells and fluorescence intensities using anatomical segmentations. However, one very important feature resulting from the alignment of raw data to a reference atlas is the three-dimensional digital map which encompasses all the spatial information relating to the distribution of the measured fluorescence signal.

## Generating Digital Brain Maps

**FIGURE 1:**
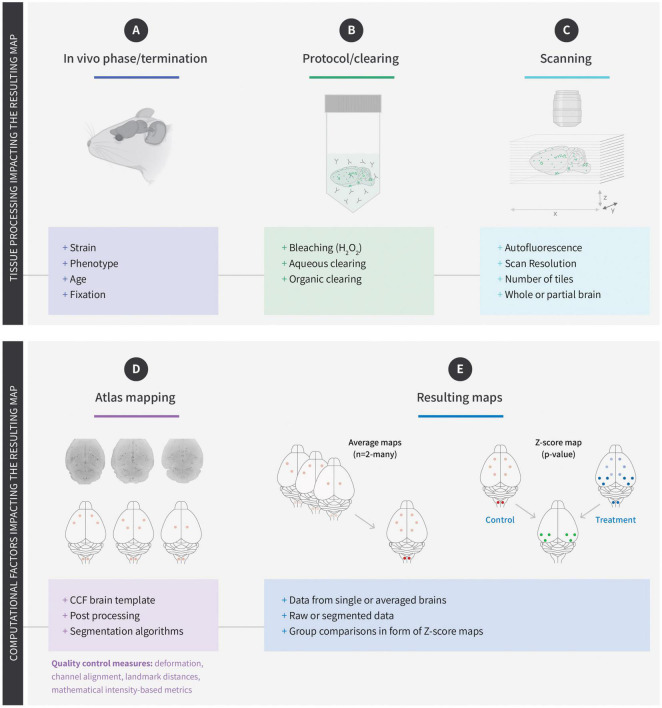
Factors influencing the generation of digital brain maps. **(A)** The choice of mouse strain can affect the overall architecture and size of the brain. Phenotypes (such as obesity) and age of the mice can lead to accumulation of lipofuscin and increased autofluorescence. The choice of fixative can impact downstream factors such as antibody penetration. Physical damage when isolating the brain may also affect subsequent registration. **(B)** The choice of protocol used for immunolabelling, and clearing is one of the biggest factors affecting registration of light-sheet fluorescence microscopy (LSFM) recorded raw data. Many factors will influence antibody penetration but also steps such as bleaching can affect autofluorescence and at the same time reduce the intensity of exogenously applied fluorescence (biodistribution). Finally, the clearing step will often lead to shrinkage or expansion of the tissue and may cause erroneous mapping. **(C)** When scanning the brains filter settings, step size, scan resolution, stitching are all factors that impact the registration to a reference brain. **(D)** The choice of CCF template and registration algorithm is important for the registration quality and should in theory be designed to match the effects of the clearing media (i.e., shrinkage or expansion). The resulting map generated from the raw data relies on the segmentation used to quantify the signal and can range from fluorescent intensities to cell nuclei or vascular segmentation. **(E)** Once the data have been mapped, they can be combined in various ways. The data can therefore be represented as single maps, average maps, or z-score (statistical) maps which encompass the data from multiple groups of animals.

The use of brain templates is common for MRI recorded data (Badea et al., 2019; Gutierrez-Barragan et al., 2022). However, for LSFM recorded data the same approach is complicated by the many different factors influencing tissue morphology and subsequent atlas registration (Figures 1A–C). In most cases, the autofluorescence is used to register the raw data to the reference brain. Consequently, factors that will influence the autofluorescence may also impact the final registration or affect the quantification of cells or signal intensities. One example is aging which leads to a buildup of lipofuscin which has been shown to increase the overall levels of autofluorescence and complicate the quantification of cFos positive cells (Perens et al., 2021a). Being an *ex vivo* technology, digital brain maps generated using LSFM depend heavily on the reagents used to clear the samples (Zhu et al., 2018). While some clearing reagents preserve the endogenous fluorescence of proteins such as GFP or RFP, others will quench it. Similarly, some clearing protocols will cause the tissue to expand while others lead to tissue shrinkage (Ueda et al., 2020; Vieites-Prado and Renier, 2021). Ultimately, the generated data is mapped to an annotated reference brain (Figure 1D; Liebmann et al., 2016; Renier et al., 2016; Salinas et al., 2018), which in most cases is the Mouse Brain Common Coordinate Framework version 3 (CCFv3) from Allen Institute of Brain Science (AIBS) (Wang et al., 2020). However, the degree of tissue distortion inferred by the clearing reagents may impact the quality of the alignment and lead to erroneous correspondence of anatomical structures between the sample and atlas. For this reason, reference atlases have been generated for specific clearing reagents (Murakami et al., 2018; Perens et al., 2021a). While these atlases improve the quality of alignment, they are not compatible with brain maps aligned to AIBS CCFv3. One way to overcome this is to develop algorithms that allow switching between different atlases (Claudi et al., 2020; Tyson and Margrie, 2022). Although these algorithms are still being developed, they will eventually enable bridging of data acquired with different imaging modalities making it possible to overlay *in vivo* images obtained with MRI and *ex vivo* data obtained with LSFM, or images acquired with other imaging techniques. A significant benefit of the digital maps generated following alignment of the raw data with a brain atlas is that their relatively small size (approx. 100 megabytes) makes them suitable for sharing and browsing in interactive online platforms. The resolution of a raw LSFM dataset depends on several parameters such as clearing protocol, type of microscope, and scan settings, but in most cases, the minimum resolution of the raw data is 1–2 μm^2^ in the x/y scanning plane and 5 μm in z-axis which corresponds to 10–12 gigabytes. However, datasets can be much bigger and multi-tiled scans obtained with high-resolution objectives may produce images in terabyte-range. In contrast, the atlases available for alignment provide an isotropic voxel size of 10–25 μm^3^ (Wang et al., 2020; Perens et al., 2021a). Consequently, the atlas-aligned maps no longer support single cell resolution, and it is not possible to study co-localization or intracellular events using such brain maps. However, the ability to combine multiple digital maps in the same spatial reference space, enables the generation of probability or statistical maps (Figure 1E). These maps are particularly useful because they encompass sample variations caused by differences in biology, tissue processing, and atlas alignment. Although individual mouse brains are similar, they are not identical, and some biological variation is to be expected. This may include subtle differences in brain morphology, gene expression, or how each animal responds to drug treatment or physiological changes (Renier et al., 2016; Hansen et al., 2021; Perens et al., 2021a). In addition, physical damage may occur when the brain is dissected or during processing. The resulting brain map from an individual mouse will therefore be affected by all these parameters. However, when the maps from several mice, treated with the same drug and processed the same way, are superimposed on top of each other, signal variations from individual mice will be diluted while the similarities will be enhanced. The resulting average maps will therefore reflect the most probable areas of gene expression or neural activity. The average maps of several study groups can also be combined to visualize differences between treatment groups (Salinas et al., 2018; Roostalu et al., 2019) or genotypes (Skovbjerg et al., 2021). When studying drug responses or other types of induced neuronal activity, it is necessary to generate voxel-wise Z-score or *p*-value maps, where the observed differences between the treatment and control groups are taken into account at voxel level (Renier et al., 2016; Nectow et al., 2017; Hansen et al., 2021; Perens et al., 2021b). A single digital Z-score map can consequently comprise information from several study groups and many mice. So, although digital brain maps lack the high resolution of the original images, they solve some of the major issues the LSFM field has been facing when trying to apply the technology to drug development, namely embedded statistical information, and reasonable sizes for cloud-based sharing. It should be noted that not all signals are equally well-represented in z-score maps. Scattered cells or random structures such as vasculature are best viewed in single maps because the large intra-group variation will lead to smearing of the signal when generating the average maps.

## Virtual Neuroscience

**FIGURE 2:**
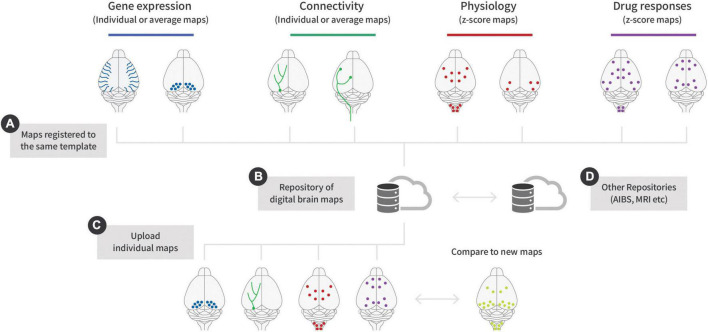
Combining different types of digital maps using repositories. **(A)** Gene expression maps can be generated from either fluorescent proteins or antibodies. Connectivity maps are usually generated using viral injections into specific neuronal populations. The virus can either be injected centrally or into peripheral organs where retrograde and synaptic transmission can label the connected brain regions. For both physiological and drug-induced neural activity it is necessary to use statistical maps to visualize the differences between treatment and control. **(B)** Data mapped to the same template can be stored in cloud-based repositories. **(C)** Individual brains maps can be uploaded and compared to new maps. For example, it is possible to compare the effects of new drugs to existing benchmarks. **(D)** In the future, it might be possible to align maps across different databases, combining *in vivo* and *ex vivo* data or overlap neuronal activation with gene expression or single cell sequencing data.

The concept of registering CNS data to an atlas was pioneered by Allen’s CCF (Allen Institute for Brain Science, 2011) and today the AIBS is used extensively by neuroscientist around the world. However, the combination of LSFM and subsequent alignment of brain samples to a reference space has enabled individual labs to produce digital brain maps. Digital brain maps can be generated from any endpoint in the brain that can be visualized using fluorescence (Figures 2A–D). At present, this includes gene expression (Renier et al., 2014; Roostalu et al., 2019; Mano et al., 2021), neuronal projections (Ueda et al., 2020; Mano et al., 2021), physiological neuronal activity (Renier et al., 2016; Nectow et al., 2017), drug-induced neuronal activity (Salinas et al., 2018; Boland et al., 2021; Hansen et al., 2021; Perens et al., 2021a; Skovbjerg et al., 2021), drug distribution (Secher et al., 2014; Gabery et al., 2020), and neurological pathologies (Liebmann et al., 2016; Detrez et al., 2019). If all the maps generated in these studies had been mapped to the same reference brain, they would, in theory, be compatible with each other (Figure 2A). Because digital brain maps are relatively small in comparison to the original datasets, they can readily be deposited in databases and browsed in real-time using cloud-based platforms and recently databases (Figure 2B) such as Cubic Cloud^1^ and NeuroPedia has been developed for this purpose^2^. Consequently, it will therefore be possible to virtually combine gene expression with neuronal projections or neuronal activity in response to a drug or physiological condition (Figure 2C). However, a major caveat of digital brain maps is that once the data has been aligned to a reference space, it is practically impossible to determine the quality of the registration and the detection of the specific endpoint. Since tissue clearing can lead to either shrinkage or expansion depending on the choice of clearing reagents, this will impact subsequent alignment differently (Murakami et al., 2018; Zhu et al., 2018; Perens et al., 2021a). A number of parameters can be measured when registering the raw data to a brain template (Figure 1D; Quality control measures). For example, it is possible to measure the degree of deformation required to align individual brains to a given atlas (Perens et al., 2021a) or to quantify changes in regional brain volumes in response to gene inactivation (Skovbjerg et al., 2021). It might also be possible to use AI algorithms to place a series of landmarks within the brain and use these to determine the quality of alignment. However, it remains to be determined if such parameters can be used to validate the overall quality of digital maps. In case of immunolabelling, the detection of specific endpoint depends on the choice of protocol, antibody penetration, antibody specificity, and microscope settings. These factors are very difficult to control for, but one solution could be to record as much metadata as possible which can then be provided together with each map. This will not eliminate maps with false information but over time several maps will show similar patterns increasing the likelihood that they are correct. Going forward it will be important to define standardized quality measures for alignment of whole brain data to reference atlases in a similar manner to those defined in other fields of research (Wilkinson et al., 2016).

## Future Perspectives and Implications in Drug Development

In previous decades several omics revolutions have taken place. Common for all of them is that they required repositories for sharing information and data. Recently digital brain maps generated from LSFM acquired data have made it possible to make similar repositories for sharing information on the brain (see text footnotes 1, 2). The kind of maps that can be generated ranges from gene expression, connectivity, neuronal activation, and pathological endpoints. However, using online browsers it will also be possible to compare all these maps interactively on a laptop. For biotech and pharma companies, this will enable them to compare the digital maps from a new drug candidate to the maps of existing drugs that are already on the market or to maps derived from drugs with known side effects. In the case of diabetes, it would be possible to use retrograde tracing to label regions in the brain responsible for innervating the beta cells in the pancreas (Rosario et al., 2016). This map could be used in combination with neural activation in response to changes in blood glucose or maps derived from models of type 1 and type 2 diabetes. In turn, these maps could be superimposed on top of drugs known to affect blood glucose. Combination of all this information may help to identify the most important regions regulating blood glucose and eventually support the development of better drugs. A similar approach could be applied to any disease originating in the brain; however, it is important to note that the maps might come from many individual labs and this collaborative effort may pave the way for community-driven virtual neuroscience. The combination of metadata and digital brain maps in shared repositories has the potential to accelerate drug discovery by allowing sophisticated data mining. A long-term ambition of LSFM recorded brain data is therefore to become more established in biotech and pharma industry and directly benefit patients suffering from CNS-derived disorders. In the future, it may even be possible to combine imaging data with other maps derived from spatial transcriptomics or single cell sequencing allowing imaging data to be correlated to gene expression (Figure 2D; Zingg et al., 2014; Erö et al., 2018; Morita et al., 2019; Reimann et al., 2019; Tiklová et al., 2019; Chen et al., 2020; Shichkova et al., 2021).

## Author Contributions

Both authors conceived and wrote the manuscript and approved the submitted version.

## Conflict of Interest

JP and JH-S were employees at Gubra and owned shares in Gubra. Gubra was the owner of NeuroPedia (www.neuropedia.dk).

## Publisher’s Note

All claims expressed in this article are solely those of the authors and do not necessarily represent those of their affiliated organizations, or those of the publisher, the editors and the reviewers. Any product that may be evaluated in this article, or claim that may be made by its manufacturer, is not guaranteed or endorsed by the publisher.

## Footnotes

1
https://cubic-cloud.com/

2
www.neuropedia.dk
